# Bootstrap Exploratory Graph Analysis of the WISC–V with a Clinical Sample

**DOI:** 10.3390/jintelligence11070137

**Published:** 2023-07-10

**Authors:** Marley W. Watkins, Stefan C. Dombrowski, Ryan J. McGill, Gary L. Canivez, Alison E. Pritchard, Lisa A. Jacobson

**Affiliations:** 1 Department of Educational Psychology, Baylor University, Waco, TX 76798, USA; 2 Department of Graduate Education, Leadership and Counseling, Rider University, Lawrenceville, NJ 08648, USA; sdombrowski@rider.edu; 3 Department of School Psychology and Counselor Education, William & Mary, Williamsburg, VA 23185, USA; 4 Department of Psychology, Eastern Illinois University, Charleston, IL 61920, USA; 5 Department of Neuropsychology, Kennedy Krieger Institute, Baltimore, MD 21231, USA; 6 Department of Psychiatry & Behavioral Sciences, Johns Hopkins School of Medicine, Baltimore, MD 21231, USA

**Keywords:** WISC–V, intelligence, dimensionality, exploratory graph analysis, construct validity

## Abstract

One important aspect of construct validity is structural validity. Structural validity refers to the degree to which scores of a psychological test are a reflection of the dimensionality of the construct being measured. A factor analysis, which assumes that unobserved latent variables are responsible for the covariation among observed test scores, has traditionally been employed to provide structural validity evidence. Factor analytic studies have variously suggested either four or five dimensions for the WISC–V and it is unlikely that any new factor analytic study will resolve this dimensional dilemma. Unlike a factor analysis, an exploratory graph analysis (EGA) does not assume a common latent cause of covariances between test scores. Rather, an EGA identifies dimensions by locating strongly connected sets of scores that form coherent sub-networks within the overall network. Accordingly, the present study employed a bootstrap EGA technique to investigate the structure of the 10 WISC–V primary subtests using a large clinical sample (*N* = 7149) with a mean age of 10.7 years and a standard deviation of 2.8 years. The resulting structure was composed of four sub-networks that paralleled the first-order factor structure reported in many studies where the fluid reasoning and visual–spatial dimensions merged into a single dimension. These results suggest that discrepant construct and scoring structures exist for the WISC–V that potentially raise serious concerns about the test interpretations of psychologists who employ the test structure preferred by the publisher.

## 1. Introduction

Surveys have consistently demonstrated that Wechsler’s scales are widely used by psychologists for making diagnostic decisions and treatment recommendations (Benson et al. 2019; Lockwood et al. 2022; Wright et al. 2017). The current Wechsler Intelligence Scale for Children—Fifth Edition (WISC–V; Wechsler 2014) was developed to measure five Cattell–Horn–Carroll (CHC; Schneider and McGrew 2018) abilities: crystalized ability (Gc), fluid reasoning (Gf), visual processing (Gv), short-term memory/working memory (Gsm/wm), and processing speed (Gs). Accordingly, its scoring structure includes five primary index scores (verbal comprehension, fluid reasoning, visual–spatial, working memory, and processing speed) that psychologists tend to use in practice (Kranzler et al. 2020; Sotelo-Dynega and Dixon 2014).

An alternative theory has been forwarded to account for the positive manifold of intelligence test batteries. This mutualism model suggests that there is a dynamic interplay between so-called field-experiences and the growth of cognitive abilities over time. For example, a child is exposed to vocabulary instruction that, in turn, produces a growth in short-term memory ability, which, in turn, augments the growth in other cognitive correlates to the point at which the positive manifold is amplified and the structure of cognitive abilities is considered solidified. However, the mutualism model was not employed in the development of the WISC–V and there is no widely accepted empirical method that can distinguish between CHC and mutualism models (Gignac 2016; Gottfredson 2016; Kan et al. 2019, 2020).

The best professional practice requires that validity evidence regarding the test content, internal structure, and relationships to other variables be considered when interpreting *all* WISC–V scores (American Educational Research Association et al. 2014). Evidence regarding the internal structure of the WISC–V is especially important because the internal structure serves as the statistical rationale for the test’s scoring structure (Braden and Niebling 2012)—that is, the degree to which scores of a test are a reflection of the dimensionality of the construct being measured. Discrepant construct and scoring structures would suggest that test scores are not “consistent with expectations regarding the construct(s) that the scale is intended to measure” (Flora and Flake 2017, p. 82), which might invalidate diagnostic decisions and treatment recommendations, particularly those espoused in treatment manuals associated with the test in question (e.g., Flanagan and Alfonso 2017).

A factor analysis, a multivariate statistical technique which assumes that unobserved latent variables cause the covariation among observed test scores (Flora and Flake 2017; Watkins 2021), has traditionally been employed to provide provisional structural validity evidence (Montgomery et al. 2018). For example, confirmatory factor analyses of the WISC–V standardization sample reported in the *Technical and Interpretive Manual* (Wechsler 2014) tacitly support the five-factor structure closely matching the CHC theoretical model, thus implying consistent construct and scoring structures for the instrument. While some researchers have replicated that result (e.g., Chen et al. 2015; Reynolds and Keith 2017), others have criticized the methodology used in those studies and found an alternative four-factor solution more consistent with the previous Wechsler theory to be more probable (e.g., Canivez et al. 2020; Canivez and Watkins 2016; Dombrowski et al. 2017, 2019, 2022; Watkins and Canivez 2022). Consequently, structural validity evidence for the WISC–V remains equivocal and its scoring structure may be inconsistent with the construct(s) it was intended to measure, thus raising questions about the veracity of the interpretive and diagnostic conclusions derived from the structure suggested by the publisher.

Given that more than two dozen factor analyses of the WISC–V have been published with standardization and clinical samples, it is unlikely that any new factor analytic study will resolve this dimensional dilemma. However, recent developments in network psychometrics offer an alternative approach for explaining correlation structures that might be useful (Borsboom 2022; Isvoranu et al. 2022). Network models focus on the direct relationships between test scores rather than assuming that a common underlying construct is responsible for the observed intercorrelations. Traditionally, network models are presented in graphs where test scores are represented by nodes connected by edges representing partial correlation coefficients between two scores after conditioning on all other test scores (Golino et al. 2022a).

Golino and Epskamp (2017) argued that clusters of strongly connected nodes, or communities, in a network would equate to the number of latent dimensions and developed exploratory graph analysis (EGA) routines based on this premise. Subsequently, a number of studies have supported the accuracy of EGA methods (Christensen and Golino 2021a; Cosemans et al. 2022; Golino and Demetriou 2017; Golino et al. 2020) as viable alternatives to traditional methods of factor extraction estimation (e.g., Horn 1965). These results, in turn, have prompted researchers to apply EGAs to data from intelligence tests (e.g., Bulut et al. 2021; McGrew et al. 2023; Neubeck et al. 2022; Schmank et al. 2021).

EGAs have been supported by statistical simulations and productively applied to data from intelligence tests. As a result, McGrew and colleagues (McGrew et al. 2023) have recently called for the greater use of these methods to aid in the understanding of the psychological structure of commercial ability measures. However, EGAs have yet to be employed with WISC–V data, which is a glaring omission given the prominent role of the test and its progenitors in the realm of intellectual assessment research and practice. Accordingly, this study employed EGAs to investigate the structure of the 10 WISC–V primary subtests in a large clinical sample.

## 2. Materials and Methods

### 2.1. Participants

The WISC–V was administered to a total of 7149 youth between the ages of 6 and 16 years as part of clinical assessments through a large outpatient pediatric psychology/neuropsychology clinic within a children’s specialty hospital in the United States. Deidentified data were retrieved from the electronic medical records of participants whose assessments included the 10 WISC–V primary subtests. The study was approved by the hospital’s Institutional Review Board.

Table 1 presents the demographic characteristics of the total clinical sample. As shown, the sample was primarily composed of White/Caucasian and Black/African American youth. The participants’ ages ranged from 6.0 to 16.93 years and averaged 10.72 years (SD = 2.77 years). Table 2 presents the clinical composition of the sample. Table 2 demonstrates that four diagnostic groups (ADHD, 48.99%; anxiety, 10.73%; adjustment disorders, 5.37%; and other nervous system disorders, e.g., encephalopathy, exposures, and non-traumatic diffuse brain dysfunction, 13.12%) comprised over three-fourths of the sample.

In terms of an a priori power estimation, Isvoranu and Epskamp (2021) reported that 1000 participants would be sufficient for generating a visual network alignment and 5000 participants would allow good estimates of the true network structure. As a result, the current sample was deemed adequate for the methodological procedures employed.

### 2.2. Instruments

The WISC–V (Wechsler 2014) contains 16 subtests in total, but its 10 primary subtest batteries are typically administered in clinical practice (Benson et al. 2019). The score structure for these primary batteries includes the verbal comprehension index derived from the similarities and vocabulary subtests, the visual spatial index produced by the block design and visual puzzles subtests, the fluid reasoning index derived from the matrix reasoning and figure weights subtests, the working memory index derived from the digit span and picture span subtests, and the processing speed index obtained from the coding and symbol search subtests. The subtest scores had means of 10 with standard deviations of 3, whereas the index scores had means of 100 with standard deviations of 15. Detailed descriptions of the WISC–V along with evidence of its reliability and validity are available in the *Technical and Interpretive Manual* (Wechsler 2014) and a series of influential interpretative books (e.g., Kaufman et al. 2016; Sattler et al. 2016).

### 2.3. Analysis

All analyses were conducted using R version 4.2.2 (R Core Team 2022) running the EGAnet package version 1.2.3 (Golino et al. 2022b) and bootnet package version 1.5 (Epskamp and Fried 2022). The analyses were guided by the tutorials provided by Christensen and Golino (2021a) and Epskamp and colleagues (Epskamp et al. 2018).

The EGA analyses consist of a sequential series of four statistical procedures, each of which may involve parameter or model estimates. First, a partial correlation matrix is computed from the original data. Second, a graphical least absolute shrinkage and selection operator (GLASSO) is used to estimate a network model, where edges are the partial correlations between the nodes, controlling for all other nodes in the network (Friedman et al. 2008). Because partial correlation networks are vulnerable to overfitting and unstable estimates (Costantini et al. 2019), the GLASSO removes spurious relationships by using a regularization technique called the least absolute shrinkage and selection operator (LASSO; Tibshirani 1996) to produce a more parsimonious network. The LASSO has a hyperparameter, λ, that controls the sparsity of the resulting network. Given the sensitivity of this hyperparameter, the usual EGA approach is to compute models across 100 or more values of λ to select the model that minimizes the extended Bayesian information criterion (EBIC; Chen and Chen 2008). The EBIC itself has a hyperparameter (γ), that controls how much it prefers models with fewer edges over models with more edges. As with the λ hyperparameter, the EGA approach is to apply several values of γ to achieve an optimally balanced network model. The default values for both λ and γ provided by the EGAnet and bootnet packages were accepted for the current study.

Third, the resulting optimized network model is analyzed for distinct communities, or strongly connected sets of nodes, using the Walktrap community detection algorithm (Pons and Latapy 2006). This algorithm assumes that the network will be globally sparse, but locally dense. That is, some nodes will be highly interconnected with each other, but exhibit few links to other nodes. A network is considered to have a good community structure when the average edge weight within a community is higher than the edge weights between that community’s nodes and the nodes in other communities (Pons and Latapy 2006). Similar to a cluster analysis, the Walktrap algorithm attempts to identify the number and composition of communities while maximizing the computational efficiency. Starting in a random node, the algorithm repeatedly moves along the edges connecting that node to its neighbors. Failing to find a node with an edge weight that exceeds the community average within *t* moves will identify a community. The resulting communities of highly correlated nodes are equivalent to dimensions (Christensen and Golino 2021a). A *t* value of 5 was applied in the current study (as per Jamison et al. 2021 and Pons and Latapy 2006).

Fourth, the original data are repeatedly resampled with replacement to generate a sampling distribution of the network using the methods described by Christensen and Golino (2021a). Their bootstrap exploratory graph analysis “approach allows for the consistency of dimensions and items to be evaluated across bootstrapped EGA results, providing information about whether the data are consistently organized in coherent dimensions or fluctuate between dimensional configurations” (Christensen and Golino 2021a, p. 481). Ten thousand bootstrap samples were generated to improve the consistency of the results (Epskamp et al. 2018) and used to estimate a typical network structure, which was formed by the median partial correlations over the 10,000 bootstraps.

## 3. Results

The summary statistics for the participants’ WISC–V scores are presented in Table 3 and the WISC–V Pearson and unregulated partial correlation matrices are provided in Table 4. Table 3 shows that the sample, when compared with the US standardization sample, was slightly below average in the subtest and composite scores, which is typical of clinical samples. All subtest and composite scores showed univariate normal distributions with no appreciable skewness or kurtosis, suggesting that they were normally distributed. Likewise, the Pearson correlations (Table 4) displayed a positive manifold with all subtests correlating positively (0.36 to 0.74).

**Figure 1:**
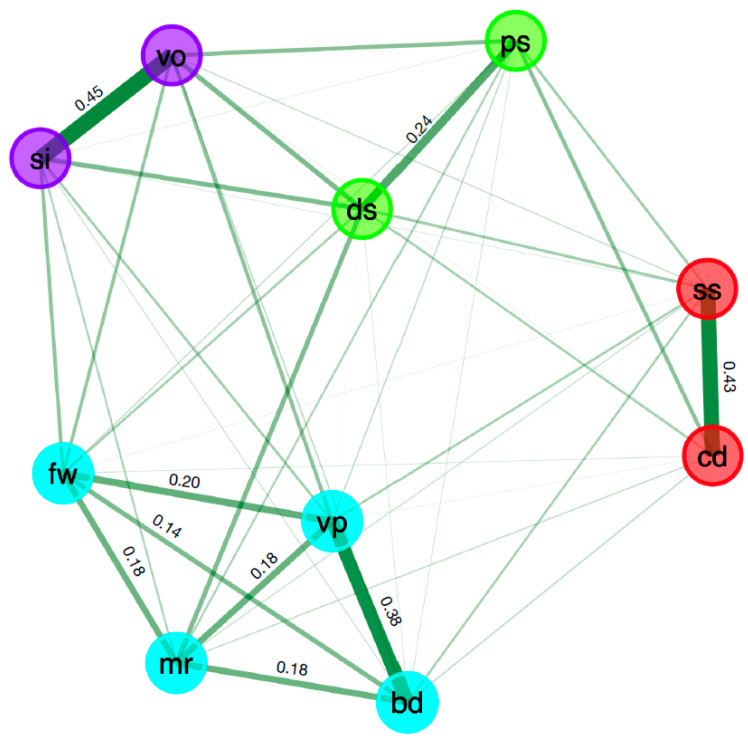
Typical network structure for the 10 primary subtests of the WISC–V with regulated edge weights for its four communities based on median partial correlations over 10,000 bootstraps. Note: bd = block design, si = similarities, mr = matrix reasoning, ds = digit span, cd = coding, vo = vocabulary, fw = figure weights, vp = visual puzzles, ps = picture span, and ss = symbol search. The color of the nodes represents the dimensions and the thickness of the lines represents the magnitude of the regulated partial correlations.

The typical network structure based on the median partial correlations over 10,000 bootstraps is presented in Figure 1. The edge weights (regulated partial correlations) within communities ranged from 0.14 to 0.45, where weights of 0.15 are small, 0.25 are moderate, and 0.35 are large (Christensen and Golino 2021b). The similarities and vocabulary nodes (verbal comprehension) and the symbol search and coding nodes (processing speed) were strongly internally connected (0.45 and 0.43, respectively), while the digit span and picture span nodes (working memory) were moderately connected, at 0.24. The fourth community merged four nodes (figure weights, matrix reasoning, block design, and visual puzzles) with small to large internal edge weights (i.e., 0.14 to 0.38), making this dimension inconstant. However, the average relationships within these four communities (e.g., 0.21 to 0.45) were superior to their relationships with the nodes in other communities (e.g., 0.04 to 0.07), indicating a good community structure according to Pons and Latapy (2006).

**Figure 2:**
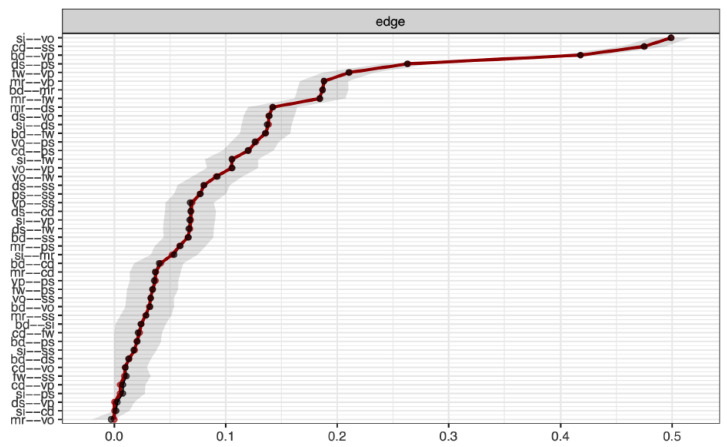
Bootstrapped confidence intervals of unregulated partial correlations for the 10 primary subtests of the WISC–V based on 10,000 bootstraps. Note: bd = block design, si = similarities, mr = matrix reasoning, ds = digit span, cd = coding, vo = vocabulary, fw = figure weights, vp = visual puzzles, ps = picture span, and ss = symbol search. The dots are point estimates of edges and the grey area depicts the bootstrap confidence intervals.

As displayed in Figure 2, the partial correlation estimates were stable. The network structure presented in Figure 1 was structurally consistent in that all four empirically derived dimensions were recovered from 100% of the bootstrap samples. Christensen and Golino (2021a) suggested that this is “an alternative yet complementary approach to internal consistency measures in the factor analytic framework” (p. 482). Additionally, this typical network structure was marked by 100% item stability. That is, each WISC–V subtest was consistently placed within the empirically derived dimensions displayed in Figure 1.

The EGA was explicitly developed to “accurately recover the number of simulated factors, presenting higher accuracy than traditional factor analytic-based methods” (Golino et al. 2022a, p. 4). Traditional dimensional methods include a parallel analysis (Horn 1965) and minimum average partials (MAP, Velicer 1976). In this case, both a parallel analysis and MAP indicated that one factor was sufficient, whereas the EGA suggested that four factors were needed. Thus, the EGA and the traditional dimensional methods were inconsistent.

Follow-up exploratory factor analyses with one and four factors were conducted (principal axis extraction and promax rotation for multiple factors following the best-practice recommendations of Watkins 2021) to ascertain the validity of these extraction criteria. The one-factor solution accounted for 50.6% of the variance and exhibited robust factor pattern coefficients ranging from 0.58 for the coding subtest to 0.80 for the visual puzzles subtest. The alpha reliability of this ten-subtest factor was 0.91 (95% CI of 0.906–0.913). The standardized root-mean-squared residual (SRMR) of this one-factor model was 0.06. However, twenty residual correlations exceeded 0.05 and four exceeded 0.10, suggesting the presence of additional factors. 

The four-factor solution paralleled the EGA network model, accounting for 65.2% of the variance. The pattern coefficients were robust (ranging from 0.52 for the picture span subtest to 0.88 for the visual puzzles subtest) and there were no salient cross-loadings. The alpha reliabilities of the factors ranged from 0.88 (95% CI: ±0.005) for the four-subtest factor to 0.72 (95% CI: ±0.014) for the factor formed by the digit span and picture span subtests. The SRMR of this model was 0.006 and the zero-residual correlations exceeded 0.05. As expected, the resulting four factors were highly correlated, with a mean of 0.67 and a standard deviation of 0.07. Partial correlations, as applied in the EGA, removed the general variance that would otherwise be extracted in a second-order factor analysis. In fact, Gorsuch (1988) maintains that “correlated factors imply the existence of higher-order factors” (p. 250). 

## 4. Discussion

The structural validity evidence for the WISC–V has been primarily investigated with factor analytic methods. A factor analysis is a multivariate statistical technique which assumes that unobserved latent variables are responsible for the covariation among observed test scores (Flora and Flake 2017; Watkins 2021). However, factor analytic results have been inconsistent. Some studies (e.g., Chen et al. 2015; Reynolds and Keith 2017) have replicated the five-factor solution that duplicates the scoring structure provided by Wechsler (2014), but other studies (e.g., Canivez et al. 2020; Canivez and Watkins 2016; Dombrowski et al. 2022; Watkins and Canivez 2022) reported that four factors could better explain the covariation among WISC–V subtests. Given the panoply of methods that have been utilized and alternative structures posited for the instrument over the course of the last decade, it is unlikely that additional factor analytic investigations will be dispositive (Dombrowski et al. 2021).

Rather than assuming that a common underlying construct is responsible for the observed intercorrelations between WISC–V subtest scores, a network psychometrics approach called an exploratory graph analysis (EGA) that focuses on the direct relationships between test scores was applied. Based on network psychometrics, these methods utilize partial correlations to detect clusters of strongly connected subtests, or communalities, in a network that would equate to the number of latent dimensions (Golino and Epskamp 2017). To ascertain the consistency of the EGA results, a bootstrap EGA with 10,000 replications was performed. The resulting median partial correlation structure consisted of four communities that mirrored the four latent factors often identified in factor analytic research (e.g., Dombrowski et al. 2022), which is more consistent with the previous Wechsler theory. While a clinical sample was used for this study, concerns regarding the generalizability of the findings for the population at large were mitigated by the similar findings from other studies on the WISC–V factor structure in nonclinical or standardized samples (Canivez and Watkins 2016; Dombrowski et al. 2017, 2019, 2022).

The CHC model of intelligence (Schneider and McGrew 2018), upon which the WISC–V is based, has been extensively investigated via factor analyses and “represents the prevailing framework by which the structure of human cognitive and intellectual abilities is understood” (Wasserman 2019, p. 249). However, there are competing theories in the intelligence literature, for example, sampling and mutualism models (Van Der Maas et al. 2017). Mutualism models conceptualize a system of dynamic reciprocal interactions between abilities that relate to each other directly, rather than through common latent variables. Some advocates contend that network models exemplify the mutualism model, and are not just an exploratory means to determine the number of factors in a dataset (Kan et al. 2019). However, research has demonstrated that latent variable and network models are statistically equivalent (Christensen and Golino 2021b; Christensen et al. 2020; Golino and Epskamp 2017; Marsman et al. 2018; van Bork et al. 2021). Given that the EGA was explicitly developed for dimensionality estimations, its application seems to be best limited, at present, to that purpose, as it is a purely exploratory method and was not designed to test any formal theoretical hypotheses. Nevertheless, some researchers seem to regard visualization methods such as psychometric network analyses as a veritable Rosetta Stone for future research on the structure of commercial ability measures such as the WISC–V (e.g., McGrew et al. 2023). However, in spite of their increasing popularity, it is important to note that critiques associated with the proliferation of the seemingly unconstrained use of these methods in psychological science have been levied (e.g., Neal et al. 2022). While it is beyond the scope of the present manuscript to fully adjudicate these matters, it is fair to say that further research on data-generating mechanisms and psychometric models will be necessary before the EGA can be confidently applied for other purposes (Christensen and Golino 2021b).

The findings of this investigation support previous research suggesting that the WISC–V measures four main constructs: language-based reasoning, visually based reasoning, working/short-term memory, and processing speed. Our findings argue against interpreting visual–spatial and fluid reasoning performances separately, and instead indicate that, at least in a clinical population, it is more appropriate to view scores in those domains as measuring the overarching construct of nonverbal reasoning. This is not to imply that visual–spatial and fluid reasoning skills are one and the same; rather, their measurement in the WISC–V does not reflect two adequately differentiable skills to warrant separate clinical interpretation.

## Figures and Tables

**Table 1:** Demographic characteristics of the clinical sample.

Race/Ethnicity	*N*	Percent	Sex	
			Female	Male
White	3685	51.5	1303	2382
Black	2057	28.8	706	1351
Hispanic	223	3.1	72	151
Multi-racial	587	8.2	201	386
Unknown/other	597	8.4	235	362
Total	7149		2517	4632
Percent			35.2	64.8

**Table 2:** Clinical diagnostic presentation of the sample.

ICD Diagnosis	*n*	Percent
ADHD	3502	48.99
Other nervous system disorders	938	13.12
Anxiety disorders	767	10.73
Adjustment disorders	384	5.37
Mood disorders	369	5.16
Epilepsy	201	2.81
Oncologic conditions	153	2.14
Disruptive behavior disorders	147	2.06
Congenital abnormalities	130	1.82
Chromosomal abnormalities	71	0.99
Autism spectrum disorders	64	0.90
Traumatic brain injury	58	0.81
Other behavioral and emotional disorders	51	0.71
Unspecified	38	0.53
Hearing loss and ear disorders	37	0.52
Cerebral palsy	36	0.50
Learning disabilities	29	0.41
Speech/language disorders	26	0.36
Tics and movement disorders	24	0.34
Endocrine and metabolic disorders	23	0.32
Intellectual disabilities	22	0.31
Blood and immune disorders	18	0.25
Cerebrovascular and cardiac disorders	17	0.24
Prenatal and newborn disorders	16	0.22
Spina bifida	14	0.20
Muscular dystrophy	8	0.11
Kidney/urinary/digestive disorders	6	0.09
Total	7149	100.00

**Table 3:** Descriptive statistics for WISC–V subtest and index scores.

Score	*n*	*M*	SD	Skewness	Kurtosis
Block Design	7149	8.7	3.4	+0.14	−0.18
Similarities	7149	9.2	3.3	+0.01	−0.04
Matrix Reasoning	7149	9.0	3.4	+0.06	−0.13
Digit Span	7149	7.9	3.1	+0.12	+0.13
Coding	7149	7.5	3.3	+0.01	−0.37
Vocabulary	7149	9.1	3.6	+0.05	−0.50
Figure Weights	7149	9.5	3.1	−0.01	−0.25
Visual Puzzles	7149	9.6	3.3	−0.03	−0.38
Picture Span	7149	8.5	3.2	+0.13	−0.16
Symbol Search	7149	8.2	3.2	+0.01	+0.01
Verbal Comprehension	7050	95.4	17.5	−0.03	−0.15
Visual–Spatial	7052	95.2	17.4	+0.10	−0.15
Fluid Reasoning	7050	95.6	16.8	+0.02	−0.36
Working Memory	7051	89.9	16.0	+0.13	−0.10
Processing Speed	7049	87.6	17.1	−0.13	−0.03
Full-Scale IQ	6647	91.0	17.4	+0.20	−0.25

**Table 4:** Pearson and unregulated partial correlations for ten WISC–V primary subtests.

	BD	SI	MR	DS	CD	VO	FW	VP	PS	SS
BD	–	0.02	0.19	0.01	0.04	0.04	0.13	0.42	0.02	0.07
SI	0.51	–	0.07	0.14	−0.01	0.51	0.10	0.06	0.00	0.02
MR	0.62	0.51	–	0.15	0.04	−0.03	0.19	0.19	0.06	0.03
DS	0.48	0.56	0.53	–	0.07	0.14	0.07	−0.01	0.26	0.08
CD	0.40	0.36	0.39	0.43	–	0.01	0.02	0.01	0.12	0.48
VO	0.52	0.74	0.49	0.58	0.38	–	0.10	0.11	0.13	0.03
FW	0.60	0.55	0.60	0.50	0.38	0.56	–	0.21	0.03	0.01
VP	0.73	0.56	0.64	0.50	0.41	0.58	0.65	–	0.04	0.07
PS	0.43	0.45	0.45	0.56	0.43	0.51	0.44	0.46	–	0.08
SS	0.45	0.40	0.42	0.45	0.63	0.42	0.41	0.46	0.44	–

Note: BD = block design, SI = similarities, MR = matrix reasoning, DS = digit span, CD = coding, VO = vocabulary, FW = figure weights, VP = visual puzzles, PS = picture span, and SS = symbol search. Unregulated partial correlations are above diagonal and Pearson correlations are below diagonal.

## Data Availability

Deidentified data may be made available by request to the sixth author, with an inter-institutional data sharing agreement.
